# Almond Skin Extracts and Chlorogenic Acid Delay Chronological Aging and Enhanced Oxidative Stress Response in Yeast

**DOI:** 10.3390/life10060080

**Published:** 2020-05-28

**Authors:** Duangjai Tungmunnithum, Malika Abid, Ahmed Elamrani, Samantha Drouet, Mohamed Addi, Christophe Hano

**Affiliations:** 1 Laboratoire de Biologie des Ligneux et des Grandes Cultures, INRAE USC1328, University of Orleans, 45067 Orléans CEDEX 2, France; samantha.drouet@univ-orleans.fr; 2 Bioactifs et Cosmetiques, CNRS GDR 3711, 45067 Orléans CEDEX 2, France; 3 Department of Pharmaceutical Botany, Faculty of Pharmacy, Mahidol University, Bangkok 10400, Thailand; duangjai.tun@mahidol.ac.th; 4 Laboratoire de Biologie des plantes et des micro-organismes, Faculté des Sciences, Université Mohamed Ier, Oujda 60000, Morocco; m.abid@ump.ac.ma (M.A.); a.elamrani1@ump.ac.ma (A.E.)

**Keywords:** aging, almond, chlorogenic acid, lipid peroxidation, mitochondria, 8-Oxo-guanine, oxidative stress, protein carbonylation, sirtuin, superoxide dismutase, yeast

## Abstract

Almond (*Prunus dulcis* (Mill.) D.A.Webb) is one of the largest nut crops in the world. Recently, phenolic compounds, mostly stored in almond skin, have been associated with much of the health-promoting behavior associated with their intake. The almond skin enriched fraction obtained from cold-pressed oil residues of the endemic Moroccan *Beldi* ecotypes is particularly rich in chlorogenic acid. In this study, both almond skin extract (AE) and chlorogenic acid (CHL) supplements, similar to traditional positive control resveratrol, significantly increased the chronological life-span of yeast compared to the untreated group. Our results showed that AE and CHL significantly reduced the production of reactive oxygen and nitrogen species (ROS/RNS), most likely due to their ability to maintain mitochondrial function during aging, as indicated by the maintenance of normal mitochondrial membrane potential in treated groups. This may be associated with the observed activation of the anti-oxidative stress response in treated yeast, which results in activation at both gene expression and enzymatic activity levels for SOD2 and SIR2, the latter being an upstream inducer of *SOD2* expression. Interestingly, the differential gene expression induction of mitochondrial *SOD2* gene at the expense of the cytosolic *SOD1* gene confirms the key role of mitochondrial function in this regulation. Furthermore, AE and CHL have contributed to the survival of yeast under UV-C-induced oxidative stress, by reducing the development of ROS/RNS, resulting in a significant reduction in cellular oxidative damage, as evidenced by decreased membrane lipid peroxidation, protein carbonyl content and 8-oxo-guanine formation in DNA. Together, these results demonstrate the interest of AE and CHL as new regulators in the chronological life-span and control of the oxidative stress response of yeast.

## 1. Introduction

Intake of fruit, vegetables, seeds, and nuts has been associated with lower risks of chronic and age-related degenerative diseases [1,2,3]. Diverse phytochemicals (i.e., plant non-nutrient compounds) such as carotenoids, phenolics or flavonoids have been related to these protective effects. Particularly, due to their numerous health benefits, interest in the consumption of nuts as a food rich in healthy nutrients has increased in recent decades, and today, almonds are among the most popular nut trees [3]. In addition to the beneficial action attributed to their specific lipid profile, almond skin is also a rich source of antioxidant phenolic compounds that have also attracted attention in recent years [3,4]. Interestingly, after cold-pressed oil extraction, most of the antioxidant phenolic compounds accumulated in the almond skin are retained in a skin-enriched by-product, the so-called cold-pressed oil residue, which makes it an attractive raw material for the extraction and recovery of these natural antioxidant phenolics [3,4,5,6]. Morocco, the world’s fourth-largest producer, has produced more than 100,000 tons of unshelled almonds in recent years. Many native almond trees (local ecotypes called *Beldi*) are still grown in the Eastern Morocco region [7,8]. These local almond ecotypes are known to accumulate higher levels of tocopherol and phenolic antioxidants compared to other varieties grown in the same region [6,8]. In the Eastern region of Morocco, almond trees occupy a surface of 26,000 ha, producing *ca* 15% of the national production out of almonds. This production generates an important part of the by-products, in particular those residues of cold-pressed almond oil, enriched with antioxidant phenolic compounds. Recently, an ultrasound-assisted extraction method was developed to obtain an almond extract from *Beldi* ecotypes rich in chlorogenic acid and other related phenolic acids [6].

Aging is a complex biological process involving multiple actors and controlled by a variety of genetic and/or environmental factors [9]. A variety of hypotheses have been suggested to explain the mechanism of aging, including the theory free radicals of aging proposed by Pr Harman in 1956 [10,11], which was certainly the most widely studied. This theory continues to be revised, and to date, it remains a sound theory for the aging process [9,10]. The theory explains that aging may be caused by the cumulative oxidative stress, leading to oxidative damage to various macromolecules (membrane lipids, proteins, DNA) within the cell, which may lead to cell death and possibly to the death of the organism [10]. This theory therefore suggests that antioxidants capable of scavenging reactive oxygen species (ROS) and/or reactive nitrogen species (RNS) are effective in delaying the aging process. Studies have shown, in good agreement, that various antioxidants of plant origin, in particular polyphenols, could have a therapeutic potential for aging and age-related diseases [12,13,14,15]. Evidence that phytochemicals such as resveratrol and quercetin have extended the lifespan of different models, acting through a well-conserved mechanism, has been identified, first in yeast and then confirmed in other models such *Caenorhabditis elegans*, *Drosophila melanogaster* and mice [12,16,17]. Yeast has emerged as an effective tool to identify anti-aging compounds [18,19]. This is not surprising given the high level of gene conservation and aging mechanism between the yeast and humans [18,20], as well as the successful identification of the candidate anti-aging test substance after their evaluation in yeast [12,13,14,15,18].

Resveratrol (RES) is the most studied anti-aging plant polyphenol, first reported to delay or mitigate aging in yeast by activation of Sir2 (silent information regulator 2) [12]. RES action on sirtuins (conserved orthologs of Sir2) has been confirmed in other models [16,17]. In yeast, SIR2 activation by RES has been proposed both gene expression level and enzyme activation [21,22,23,24]. However, the exact mechanism accounting for the putative longevity effects of RES is still debated [25]. Sirtuins are a conserved family of nicotinamide adenine dinucleotide (NAD+)-dependent protein deacetylases, and interestingly some compelling evidence has linked their action to ROS and aging, in particular to the ROS-driven mitochondria-mediated hormetic response [26]. Other potential anti-aging plant polyphenols and their mechanisms of action at the molecular level should be investigated. Such studies may provide important information for the use and development of anti-aging plant sources and derived compounds, and may reveal mechanisms to pave the way for anti-aging drug development. The present study demonstrated the impact of an extract from almond skin and its main component, chlorogenic acid, on the lifespan extension in yeast, and described their actions on oxidative stress.

## 2. Materials and Methods 

### 2.1. Chemicals

All solvents used in this study were of an analytical grade (Thermo Scientific, Illkirch, France). Chlorogenic acid (CHL) and resveratrol (RES) standard was purchased from LGC Standard (Molsheim, France). Other chemicals and reagents have been purchased from Sigma-Aldrich (Saint-Quentin Fallavier, France). Note that, for each assay described hereafter, the possible interfering intrinsic absorbance, fluorescence and fluorescence quenching of each compound/extract were considered and deduced from the measurements.

### 2.2. Extract Preparation

Almond oil residues were obtained from Moroccan almonds (Beldi local ecotypes) grown in Ain Sfa (34°46’42.4" N, 002°09’28.9’ W), a pilot site located in Eastern Morocco. Almond trees grown under the conditions described by Melhaoui et al. [8]. The almonds were then crushed using an oil screw press (KOMET DD85G, IBG Monforts Oekotec GmbH & Co. KG, Monchengladbach, Germany) and the resulting residue was ground to approximately 100–150 μm of particulate using a blender equipped with rotating blades (Grindomix GM 200 blender, Retsch France, Eragny, France). This material was further subjected to an optimized and validated ultrasound-assisted extraction protocol [6]. Almond skin extract (AE) was obtained by ultrasound-assisted extraction completed in an ultrasonic bath (USC1200TH, Prolabo, Sion, Switzerland) consisting of a tank with an internal dimension of 300 × 240 × 200 mm^3^ with an electrical power of 400 W corresponding to an acoustic power of 1 W/cm^2^ and a maximum heating power of 400 W. Sample was placed in 50-mL quartz tubes equipped with a vapor condenser and was suspended in 10 mL of aqueous EtOH 53.0% (v/v) as extraction solvent using a liquid-to-solid ratio of 10:1 mL/g DW (dry weight). During extraction, optimized and validated conditions with a US frequency of 27.0 kHz during 29.4 min and an extraction temperature of 45 °C were applied [6]. A similar extraction protocol was also used to quantify CHL and RES in yeast cells and the culture medium. 

### 2.3. HPLC Analysis

After extraction, each extract was centrifuged at 3000 rpm for 15 min and the resultant supernatant was filtered with a syringe filter (0.45 µm, Millipore, Molsheim, France) prior to HPLC analysis. Separation was done by HPLC (High-Performance Liquid Chromatography) by the use of a complete Varian HPLC system consisting of: Prostar 230 pump, Metachem Degasit, Prostar 410 autosampler, Prostar 335 Photodiode Array Detector (PAD) and driven by Galaxie version 1.9.3.2 software (Varian, Les Ulis, France). An RP18 column (Purospher RP-18; 250 × 4.0 mm^2^; internal diameter: 5 µm; Merck Chemicals, Molsheim, France) was used for separation at a temperature of 35 °C. The mobile phase consisted of a mixture of two solvents A (HPLC grade water with 0.2% (v/v) acetic acid) and B (HPLC grade methanol). The non-linear gradient applied for separation was: 8% B (0 min), 12% B (11 min), 30% B (17 min), 33% B (28 min), 100% B (30–35 min), 8% B (36 min) at a flow rate of 1 mL/min. A re-equilibrating time of 10 minutes was applied between each injection. Compound detection was set at 295 and 325 nm (corresponding to the λmax of the main compounds) [6].

### 2.4. Yeast Culture Conditions

Yeast strain DBY746 (*MATα leu*2-3,112 *his*3Δ1 *trp*1-289a *ura*3-52 GAI+) was used. The culture was initiated from frozen stock plated onto a yeast extract peptone dextrose (YPD) medium (Sigma-Aldrich, Saint-Quentin Fallavier, France). 

For lifespan assay, after incubation at 30 °C for 2–3 days, a single colony was incubated into 1 mL of SDC (synthetic complete dextrose) medium [27] and incubated overnight with shaking (220 rpm) at 30 °C. The overnight culture was then diluted into approximately 10 mL of fresh SDC medium to an absorbance value of 600 nm of 0.1 and incubated with shaking (220 rpm) at 30 °C. This time point is considered to be day 0 of chronological aging. Yeast chronological life-span (CSL) assay in liquid culture was used as described by Hu et al. [27]. Briefly, starting on day 3, aliquots of 10 μL were removed from the flask, diluted 10,000 times in sterile water, 10 μL of diluted culture were placed on YPD plates, incubated at 30 °C for 2–3 days, and the colony-forming unit (CFU) numbers were determined. The microcolonies formed on the YPD plates were observed under a microscope, and the daughter cells were quantified. The CFU number at day 3 of non-treated cells is considered to be the 100% survival. CHL (at 5 (CHL5), 10 (CHL10) and 25 (CHL25) µM final concentration, respectively) AE (*ca* 1 mg/mL dry extract corresponding to 25 µM of CHL final concentration), as well as positive control RES (10 µM final concentration) were dissolved in cell culture grade dimethyl sulfoxide (DMSO; Sigma-Aldrich, Saint-Quentin Fallavier, France). Final DMSO concentration was 0.1% (v/v). Control yeast was inoculated with the same DMSO concentration.

Growth index was expressed as the ratio of absorbance 600 nm of yeast cultures determined at 0 and 48 h after treatment, respectively. 

For oxidative stress assay, UV treatment was used to induce oxidative stress as described by Garros et al. [28]. In brief, yeast cells were, first, treated as described above using the same conditions (CTL, RES, AE, CHL5, CHL10 and CHL25). Six hours after extract/compound addition, yeast cells were rinsed with PBS 1X to eliminate non-absorbed compounds, and then irradiated with a UV dose of 106.5 J/m^2^ UV-C (254 nm) under a Vilber VL-6.C filtered lamp (Thermo Fisher Scientific, Villebon-sur-Yvette, France), and then incubated at 28 °C with orbital shaking at 120 rpm in the dark in complete 2.0% (w/v) glucose YPD medium (Sigma Aldrich, Saint-Quentin Fallavier, France) prior to analysis. Non-irradiated cells were grown under the same conditions. Irradiation was considered to be hour 0 of oxidative stress assay. 

### 2.5. Reactive Oxygen and Nitrogen Species Measurement

The dihydrorhodamine-123 (DHR-123) fluorescent dye (Sigma-Aldrich, Saint-Quentin Fallavier, France) was used to determine the level of reactive oxygen and nitrogen species as described by Nazir et al. [29]. Approximately 10^8^ yeast cells grown in the presence of AE, CHL or RES or DMSO (control cells) were washed twice with PBS, and then resuspended in PBS containing 0.4 μM DHR-123 and incubated during 10 min in the dark at 30 °C. After washing with PBS twice, the fluorescence signal (λex = 505 nm, λem = 535 nm) was detected using the VersaFluor Fluorimeter (Biorad, Marnes-la-Coquette, France).

### 2.6. Estimation of NAD and NADH Contents

Measurement of the NAD and NADH nucleotides was performed as described previously by Lin et al. [30] using ca 10^7^ cells. In brief, after NAD and NADH extraction by acidic and alkali extraction, respectively, followed by neutralization, enzymatic cycling reaction was performed as described by Lin et al. [30], and their concentrations determined fluorometrically with excitation at 365 nm and emission at 460 nm using the VersaFluor Fluorimeter (Biorad, Marnes-la-Coquette, France) and with the help of standard curves (0–40 µM).

### 2.7. Mitochondria Membrane Potential Evaluation

Mitochondria membrane potential (ΔΨm) was measured by monitoring the fluorescence of the specific probe 3,3′-dihexyloxacarbocyanine iodide (DiOC6(3); Sigma-Aldrich, Saint-Quentin Fallavier, France) as described by Hano et al. [31]. DiOC6(3) stains mitochondria depending on their ΔΨm [32]. Cells were incubated in culture medium with 25 nM of DiOC6(3) for 45 min at 30 °C. their fluorescence signal (λex = 482 nm, λem = 504 nm) was measured using VersaFluor Fluorimeter (Biorad, Marnes-la-Coquette, France). At least six independent measurements were performed for each condition and the results were expressed as relative fluorescent units.

### 2.8. Gene Expression by RT-qPCR Analysis

Total RNAs were extracted from the yeast cells at their exponential phase using the RiboPure RNA extraction kit (Thermo Scientific, Illkirch, France). Reverse transcription was performed using SuperScript IV cDNA synthesis kit (Thermo Scientific, Illkirch, France) with oligo (dT) adaptor primer (Thermo Scientific, Illkirch, France), 1 unit of RiboLock (Thermo Scientific, Illkirch, France) and 5 mg of yeast total RNA quantified by Quant-iT HR RNA assay and using Qubit fluorimeter (Thermo Scientific, Illkirch, France). Real-time PCR was performed with a PikoReal™ Real-Time PCR System (Thermo Scientific, Illkirch, France) using DyNAmo ColorFlash SYBR Green qPCR (Thermo Scientific, Illkirch, France) and specific primers. Primers used were: *SOD1*, forward: 5′-CACCATTTTCGTCCGTCTTT-3′, and reverse: 5′-TGGTTGTGTCTCTGCTGGTC-3′; *SOD2*, forward: 5′-CTCCGGTCAAATCAACGAAT-3′, and reverse: 5′-CCTTGGCCAGAAGATCTGAG-3′; *SIR2*, forward: 5′-CGTTCCCCAAGTCCTGATTA-3′, and reverse: 5′- CCACATTTTTGGGCTACCAT-3′; *TUB1*, forward: 5′-CCAAGGGCTATTTACGTGGA-3′, and reverse: 5′-GGTGTAATGGCCTCTTGCAT-3′. The qPCR parameters were as follows: an initial denaturation at 95 °C for 5 min, then 40 three-step cycles of 94 °C for 15 s, primer annealing at 55.4 °C for 10 s, and extension at 72 °C for 20 s. After 40 cycles, a final extension phase was carried out for 90 s at 72 °C. Observation of a single peak in the melting curve obtained after amplification indicated the existence of a single amplicon. The amounts of mRNA *SIR2*, *SOD1* and *SOD2* were normalized to that of *TUB1*. Expression levels were calculated and normalized using 2^−ΔΔCt^ method. Reactions were made in four biological replicates, and two technical replicates were performed for each measurement.

### 2.9. Enzymatic SIRT1/SIR2 and Total SOD Activities Determinations

For protein extraction, approximately 10^8^ yeast cells have been washed three times with PBS. Then, 1 mL of PBS was added and the mixture was subjected to three freeze and thaw cycles using liquid nitrogen. The cell lysate was then centrifuged at 10,000 g at 4 °C for 15 min, and the supernatant was used to prepare the sample solution by dilution with PBS. Proteins were quantified using the Qubit Protein Assay Kit following the manufacturer’s instructions and using the Qubit fluorimeter (Thermo Scientific, Illkirch, France).

Total SOD activity was measured using the Superoxide Dismutase Activity kit following the manufacturer’s instructions (Thermo Scientific, Illkirch, France).

SIRT1/SIR2 activity was determined using the SIRT1 Assay Kit (Sigma-Aldrich, Saint-Quentin Fallavier, France) following the manufacturer’s instructions and using a Versafluor fluorimeter (Biorad, Marnes-la-Coquette, France).

### 2.10. UV-Induced Oxidative Stress and Survival (Cell Viability) to Oxidative Stress Evaluation

UV-induced oxidative stress in yeast strain DBY746 (*MATα leu*2-3,112 *his*3Δ1 *trp*1-289a *ura*3-52 GAI+) grown on YPD medium was obtained as described by Nazir et al., [29]. Yeast cells were irradiated with 106.5 J/m^2^ UV-C (254 nm) under a Vilber VL-6.C filtered lamp (Thermo Fisher Scientific, Villebon-sur-Yvette, France). After overnight incubation at 30 °C, yeast cells were subjected to survival assay and further analyses. Survival was estimated by counting the colony forming unit as described in paragraph 2.4.

### 2.11. Membrane Lipid Peroxidation Evaluation

The thiobarbituric acid (TBA; Sigma Aldrich, Saint-Quentin Fallavier, France) method described by Garros et al. [28] was used for the measurement of membrane lipid peroxide. Briefly, about 10^8^ yeast cells ground in double distilled water were centrifuged for 10 min at 10,000 xg. The supernatant (75 μL) was mixed with 25 μL of SDS (3% (w/v)), 50 μL of TBA (3% (w/v) prepared in 50 mM of NaOH) and 50 µL of HCl (23% (v/v)). Mixing was performed between each addition. The final mixture was heated for 20 min at 80 °C, cooled on ice, and the absorbance was measured at 532 nm (A532). Non-specific absorbance measured at 600 nm (A600) was subtracted. Standard curve was prepared using 1,1,3,3, tetramethoxypropane to measure concentrations of TBARS in the samples.

### 2.12. Protein Carbonylation Level Estimation

Total proteins were extracted from about 10^8^ yeast cells as described in paragraph 2.8. Protein carbonyl content was determined using Protein Carbonyl ELISA kit following the manufacturer’s instructions (Cell BioLabs, Paris, France). 

### 2.13. 8-oxo-guanine Level Estimation

DNA was extracted from about 10^8^ yeast cells with Yeast DNA Extraction Reagent Kit following manufacturer’s instructions (Thermo Scientific, Illkirch, France) and 8-oxo-guanine content was determined with the 8-OHdG DNA Damage ELISA kit following the manufacturer’s instructions (Cell BioLabs, Paris, France). 

### 2.14. Statistical Analysis 

Results are expressed as means and standard deviations of at least four separate (biologically independent) replicates were used to present the data. Significant differences between groups in all experiments were determined by ANOVA, followed by two-tailed multiple *t*-tests with Bonferroni correction performed with XL-STAT 2019 biostatistics software (Addinsoft, Paris, France). All results were considered significant at *p* < 0.05.

## 3. Results and Discussion

### 3.1. Yeast Lifespan Extension induced by Almond Skin Extract Chlorogenic Acid is Accompanied by a Reduction in Reactive Oxygen/Nitrogen Species 

In order to evaluate the potential anti-aging effects of almond extract and chlorogenic acid, we followed the chronological aging of wild type yeast (Saccharomyces cerevisiae, strain DBY746) previously used for this purpose [33]. This wild type strain has a short mean life-span of less than 7 days when aged in the SDC medium [33], which is advantageous for studying the impact of plant extracts on the life span of the yeast. High purity commercial chlorogenic acid (CHL) standard was used instead of purifying it to avoid purity problems and possible cross-contamination. CHL was tested at three different concentrations: 5, 10 and 25 μM. The almond skin extract (AE) was prepared from cold-pressed oil residues of a local Beldi ecotype from Eastern Morocco rich in chlorogenic acid following an optimized ultrasound-assisted extraction procedure [6]. This AE was used as a raw material to evaluate the potential anti-aging action of almond skin phenols, but also to detect possible synergistic or antagonistic effects with chlorogenic acid due to the presence of other phenolics in the extract. The HPLC chromatogram and phytochemical characterizations of this extract are shown in Figure S1 and Table S1, respectively. In addition to chlorogenic acid, this AE also contained substantial amounts of protocatechuic acid, p-hydroxybenzoic acid and p-coumaric acid. The AE was tested at a final concentration of 1 mg/mL corresponding to a concentration of approximately 25 μM of chlorogenic acid corresponding to the highest evaluated CHL concentration. Prior to their application to yeast cells to assess their impact on life-span, the absence of any significant growth and viability effects of AE and CHL at their respective tested concentrations was evaluated in order to avoid any bias resulting from possible toxic or antifungal activities (Table S2).

**Figure 1:**
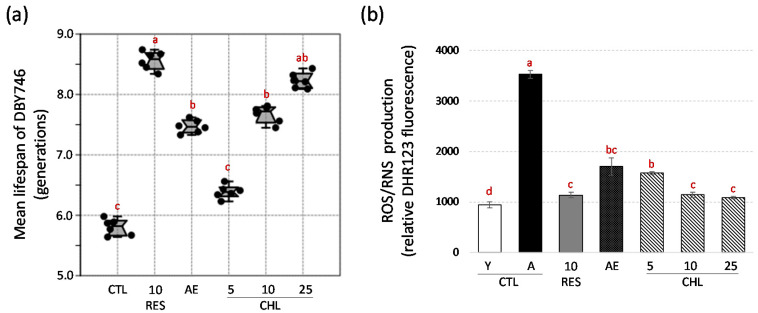
(**a**) Mean of chronological lifespan yeast (strain DBY746) calculated using the survival plots presented in Figure S2. Percentage of viable cells was determined by the microcolony method on yeast extract peptone dextrose (YPD) plates as described by Hu et al. [27]. (**b**) Production of reactive oxygen and nitrogen species (ROS/RNS) during aging process evaluated in young yeast (Y CTL) (on day 2 of cultivation) and aged yeast (A CTL) (on day 5 of cultivation) in absence or in presence of almond extract (AE, 1 mg/mL) or chlorogenic acid at 3 concentrations (CHL5, CHL10 and CHL25 corresponding to chlorogenic acid addition at 5, 10 and 25 µM, respectively). *E*-Resveratrol (RES, 10 µM) was used as control anti-aging drug. ROS/RNS production was evaluated using the dihydrorhodamine 123 (DHR123) probe. Values are means ± standard deviations (SD) of 6 independent experiments. Different letters represent significant differences between the various extraction conditions (*p* < 0.05).

The results of the chronological life-span test of the DBY746 strain of yeast are shown in Figure 1. Control cells of yeast have been inoculated with the same volume of DMSO. Resveratrol (RES) was used as a positive control at a classically applied concentration of 10 μM [34,35,36,37]. Lifespan has been evaluated with the survival plots corresponding to each treatment used to determine chronological lifespan of yeast (strain DB746) presented in Figure S2. Percentage of viable cells was determined by the microcolony method on YPD plates as described by Hu et al. [27]. Interestingly, this in situ viability assay allowing to determin the chronological life-span was described as closely related to the yeast replicative lifespan [27] and thus provides a good estimation of this parameter.

Here, the mean life-span of the CTL DBY746 yeast aged in the SDC was 5.84 ± 0.16 days. As expected, there were clearly positive effects of RES on the DBY746 chronological life-span raising it to 8.49 ± 0.17 days (Figure 1a). AE (7.44 ± 0.16 days) as well as CHL at doses of 10 (7.60 ± 0.17 days) and 25 (8.15 ± 0.08 days) µM significantly extended the chronological lifespan of the yeast strain (Figure 1a). CHL at a dose of 5 μM was not sufficient to significantly affect the lifespan of the yeast strain (Figure 1a). Given the concentration of CHL present in EA, and although the almond extract has anti-aging activity, these results may suggest the presence of antagonistic compounds in this extract, which may hinder the action of CHL. Interestingly, this result is consistent with previous observations that, although some supplements may be effective, the majority of evidence has shown that either simple or complex combinations of supplements are mostly ineffective in preventing the occurrence or progression of major causes of disease [38]. 

Here, AE increased the lifespan of yeast by 27.5%, while a dose-dependent effect was observed for CHL and a concentration of 25 µM was necessary to reach a life-span extension similar to that observed with the RES positive control (Figure 1a). The difference in concentration between the RES and the CHL required to achieve the same effect may result from a slightly lower CHL efficiency. However, it may also be the consequence of a lower absorption of CHL compared to RES. The bioavailability of a natural product is of paramount importance so that it can also effectively play its role in the cell. Absorption of RES has been reported to be highly effective in various models, including animal models and humans [39]. To have an idea about the absorption of RES vs. CHL, we measured their respective concentrations in the yeast cells as well as in the culture medium 6 hours after their addition. Here, six hours after their addition, RES was absorbed more effectively by yeasts than by CHL (Table S3). This could indicate that part of the difference observed for the dose levels needed to achieve a similar increase in lifespan for these two compounds is likely to be based on this difference in absorption. It must also be considered, however, that part of the amount of compound that could bind to the cell wall of the yeast and therefore not be absorbed effectively but still be measured in the extracts. 

Few data are available on absorption kinetics in yeast and, more specifically, on transport proteins that could be involved in RES or CHL uptake. In view of its polyhydric alcohol nature, such as arabinose, bacterial arabinose–H^+^ transport protein araE has been proposed to be able to transport RES [40]. Expressed in yeast, this transporter enhanced the accumulation of RES, but without transporting it directly [41]. Similar polyol transporters involved in the stress response have been deciphered in yeast [42] and a similar mechanism involving endogenous protein transporters could be involved in the accumulation of RES. To date, no protein transporters for CHL uptake in yeast have been described. In addition to the involvement of protein transporters, passive transport could also be considered, since passive encapsulation of RES and CHL in yeast cells acting rapidly over 4 hours of incubation has been reported [43,44]. Absorption (i.e., encapsulation efficiency in that case) varied according to the concentration in the external medium and the purity of the compound, as well as the temperature, indicating that the process could occur through passive diffusion [43,44]. In line with our data, the authors reported a passive uptake efficiency of RES higher than that of CHL [43,44]. 

ROS and RNS are metabolism by-products that are physiologically and continuously generated in mitochondria. Oxidative alterations to biomolecules increase with age, and are an obvious outcome of redox imbalance [26,45]. CHL is a phenolic compound that has antioxidant effects [6]. Because there was no difference in the NAD^+^/NAD(H) ratio estimated as described by Lin et al. [46] (Table S4), we considered that CHL and AE could extend the chronological lifespan of the yeast through antioxidative action. The production of reactive oxygen and nitrogen species (ROS and RNS) was therefore evaluated using the dihydrorhodamine 123 (DHR123) probe (Figure 1b). ROS and RNS production increased during aging when yeast was aged (on day 5 of cultivation) compared to young yeast (on day 2 of cultivation) (Figure 1b). As observed for RES, AE and CHL treatments resulted in only moderate ROS and RNS production, which at day 5 was only slightly higher than the level measured in young yeast culture. At the same time, mitochondria membrane potential (ΔΨm) evaluation, using 3-3′-dihexyloxacarbocyanineiodide (DiOC_6_(3)), suggested a loss of mitochondrial function in aged yeasts, while both AE and CHL were able to maintain a functional ΔΨm value as for young yeast (Figure S3). A similar effect has been observed for RES (Figure S3). These results suggested that AE and CHL might act by controlling the mitochondrial-mediated aging process of ROS/RNS. This hypothesis was further tested hereafter. Apple extracts (containing CHL) have been reported to increase yeast lifespan by reducing the levels of reactive oxygen species and cell sensitivity to oxidative stress through a mechanism involving mitochondria [47,48].

### 3.2. Almond Skin Extract and Chlorogenic Acid Activated Expression of Genes Involved in Oxidative Stress Resistance

Our next goal was to decipher the molecular mechanism underlying the action of AE and CHL on the life-span of the yeast. In particular, their effects on both the silent information regulator 2 (SIR2) and the superoxide dismutases (SOD1 and SOD2) steady-state mRNA levels and corresponding enzyme activity were considered. SIR2 belongs to a conserved family of Nicotinamide Adenine Dinucleotide (NAD^+^)-dependent protein deacetylases and convincing evidence has connected its activity with ROS and aging, in particular with ROS-driven mitochondrial-mediated response [26]. RES was one of the first recorded plant polyphenols to delay or reduce yeast aging through activation of SIR2 activity [12]. SODs encoded for the antioxidant stress genes which are involved in ROS scavenging. SOD1 is a Cu/Zn-SOD localized in the cytoplasm, while SOD2 is a mitochondrial Mn-SOD. SOD2 is an effective ROS scavenger, playing an important role in antioxidant response [49], and has been correlated with the control of lifespan in yeast [50].

**Figure 2:**
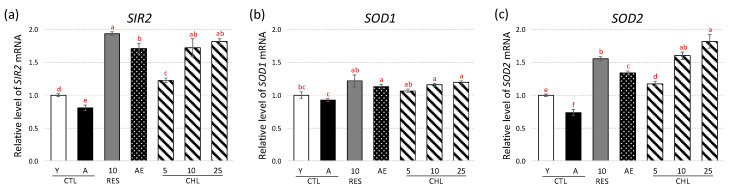
Effects of almond skin extract (AE) and CHL on SIR2 (**a**), SOD1 (**b**) and SOD2 (**c**) gene expression determined by RT-qPCR. Expression was normalized with TUB1 gene and expressed relative to young yeast cells (Y). Y CTL was young yeast (on day 2 of cultivation) and A CTL was aged yeast (on day 5 of cultivation). AE, almond extract (1 mg/mL). CHL: chlorogenic acid at 3 concentrations (CHL5, CHL10 and CHL25 corresponding to chlorogenic acid addition at 5, 10 and 25 µM, respectively). E-Resveratrol (RES, 10 µM) was used as control antiaging drug. Values are means ± standard deviations (SD) of 4 independent experiments. Different letters represent significant differences between the various extraction conditions (*p* < 0.05).

First, the steady-state mRNA level of these genes was monitored by RT-qPCR, as shown in Figure 2.

Aged yeast showed a significantly lower expression of the *SIR2*, *SOD1* and *SOD2* genes compared to young yeast, whereas, as already observed, the positive control RES could reverse that trend [34,35,36,37]. The expression of the *SIR2*, *SOD1* and *SOD2* genes was also significantly increased by AE and CHL. CHL notably induced an activation of the *SIR2* gene expression similar to that of RES (Figure 2a). A very slight induction of *SOD1* gene expression was observed (Figure 2b), while the expression of the *SOD2* gene showed a more notable increase in the presence of AE and CHL (Figure 2c). As for the expression of the *SIR2* gene, a dose-dependent response was observed with CHL. Especially, CHL induced the expression of the *SOD2* gene significantly more than RES.

**Figure 3:**
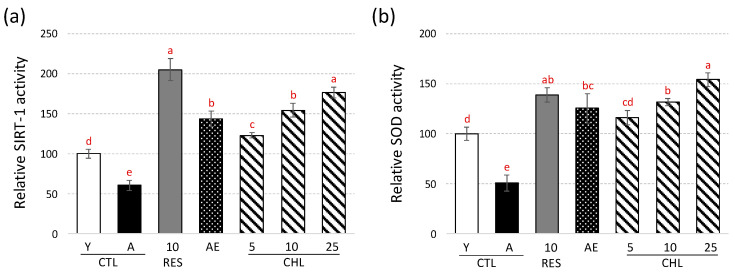
Effects of AE and CHL on SIRT1/SIR2 (**a**) and total SOD (**b**) enzyme activities. Y-CTL was young yeast (on day 2 of cultivation) and A-CTL was aged yeast (on day 5 of cultivation). AE, almond extract (1 mg/mL). CHL: chlorogenic acid at 3 concentrations (CHL5, CHL10 and CHL25 corresponding to chlorogenic acid addition at 5, 10 and 25 µM, respectively). E-Resveratrol (RES, 10 µM) was used as control antiaging drug. In young yeast culture (Y), actual SIRT1/SIR2 enzyme activity was equal to 4564.4 FU/mg protein, whereas total SOD activity was 35.6 units/mg protein. The enzyme activity was expressed as a percentage of Y-CTL. Values are means ± standard deviations (SD) of 4 independent experiments. Different letters represent significant differences between the various extraction conditions (*p* < 0.05).

In addition, the SIRT1/SIR2 and SOD assays were used to determine the effect on both SIRT1/SIR2 enzyme activities (Figure 3).

The activation of *SIR2* gene expression was further confirmed at enzyme level using a commercial SIRT1/SIR2 assay kit (Figure 3a). Aged yeast had a low SIRT1/SIR2 activity, while both young and RES treated yeast showed higher significantly SIRT1/SIR2 activity. In the same way, a 43.5% increase in SIRT1/SIR2 activity was measured in the presence of AE, in line with the RT-qPCR analysis. A dose-dependent increase (up to 76.5%) in SIRT1/SIR2 activity was observed in the presence of CHL, resulting in statistically identical activation to that observed in RES. Similarly, a comparable activation profile with total SOD activity was observed (Figure 3b), with an even more pronounced activation observed for AE and CHL (up to 81.8% increase for CHL at a dose of 25 μM). It can also be noted that the observed increase in total SOD activity was more consistent with mitochondrial *SOD2* gene expression than with mitochondrial *SOD1* gene expression. Again, an important role of mitochondria can be assumed in this regulation.

Phloridzin, an apple polyphenol, an apple flavonoid, has previously been shown to stimulate the expression of both the genes *SOD1* and *SOD2* [21]. However, for the majority of phytochemicals, including the *Citrus*-derived flavonoid hesperidin, a specific induction of *SOD2* gene expression was recorded without significant (or only moderate) activation of the *SOD1* gene expression [22,23]. This gene expression activation may result from an effective increase in SIRT1/SIR2 [21,22,23]. In various models, SIRT1/SIR2 have been shown to induce *SOD2* gene expression through deacetylation process resulting in the activation both PGC-1α and FOXO [48,51]. In yeast in particular, this resulted in the activation of *SOD2* gene expression by various plant-derived natural products and was correlated with the extension of life span [21,22,23]. Here, this increased *SOD2* gene expression may also be correlated with mitochondrial function, with both AE and CHL treatments resulting in the ΔΨm functional value being retained (Figure S3). This observation is in line with the literature data showing the role of SOD2 as an effective ROS scavenger in the antioxidant response [49] as well as in the control of lifespan in yeast [50], and it may suggest that AE and CHL can extend the lifespan of the yeast by triggering SIR2/SOD2 actions. In other models, CHL has been reported to activate the FOXO transcription factors, resulting in an increase in the lifespan of *C. elegans* [52], which is known to be regulated by SIRT1/SIR2 activity [48,51]. In humans, CHL has been shown to reverse the aging effect on cognitive functions, in particular by improving the attention, performance and memory functions of elderly people [53]. However, the molecular mechanism is still unknown, even if the antioxidant activity of CHL can be assumed. In particular, future work with genetic mutants will be conducted to test the causality of the involvement of SIR2 and/or SOD2. 

### 3.3. Almond Skin Extract and Chlorogenic Acid Increased Yeast Survival to Oxidative Stress induced by UV-C and Reduced Oxidative Cell Damages

**Figure 4:**
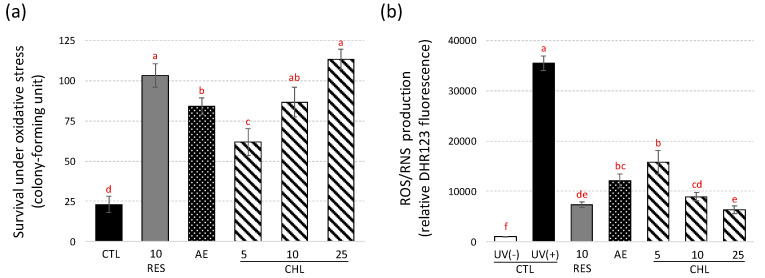
Survival and ROS/RNS production under UV-C-induced oxidative stress conditions. (**a**) Survival assay, plates were incubated at 28 °C for 3 d and the number of colonies was counted; excepted if mentioned CTL was DBY746 yeast subjected to UV-C stress. (**b**) ROS/RNS production was evaluated using the dihydrorhodamine 123 (DHR123) probe. AE, almond extract (1 mg/mL). CHL: chlorogenic acid at 3 concentrations (CHL5, CHL10 and CHL25 corresponding to chlorogenic acid addition at 5, 10 and 25 µM, respectively). E-Resveratrol (RES, 10 µM) was used as control antiaging drug. Values are means ± standard deviations (SD) of 6 independent experiments. Different letters represent significant differences between the various extraction conditions (*p* < 0.05).

Overall, the previous results suggest that AE and CHL may extend the chronological lifespan of the yeast through an antioxidant mechanism. The impact of UV-C-induced oxidative stress on DBY746 yeast was examined to test this hypothesis using our published protocol [6,28,29]. The resulting yeast survival, estimated with a quantitative colony counting assay [54], and ROS/RNS production, are presented in Figure 4. 

Under these oxidative conditions induced by UV-C, AE and CHL supplementations significantly improved the survival of yeast (Figure 4a) in conjunction with their ability to significantly reduce ROS/RNS production (Figure 4b). The protection effect of CHL against oxidative stress was within the range of RES. A similar assay, but inducing oxidative stress by H_2_O_2_, was used to assess the anti-aging action of nolinospiroside F and phloridzin in yeast [21,23]. 

**Figure 5:**
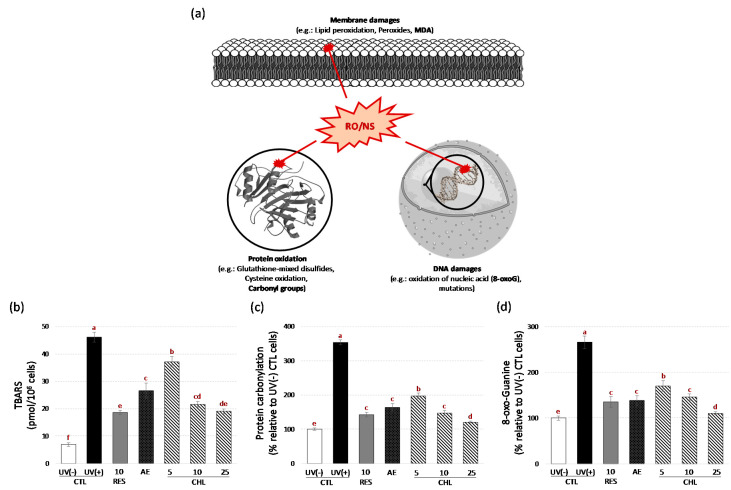
Schematic view of the different ROS/RNS-induced oxidative damages to membrane lipids, proteins and DNA (**a**), and effects of AE and CHL on malondialdehyde (MDA) levels measured by TBARS assay (**b**), protein carbonyl contents determined by ELISA assay (**c**) and 8-oxo-Guanine formation determined by ELISA assay (**d**). AE, almond extract (1 mg/mL). CHL: chlorogenic acid at 3 concentrations (CHL5, CHL10 and CHL25 corresponding to chlorogenic acid addition at 5, 10 and 25 µM, respectively). E-Resveratrol (RES, 10 µM) was used as control antiaging drug. Values are means ± standard deviations (SD) of 6 independent experiments. Different letters represent significant differences between the various extraction conditions (*p* < 0.05).

These results were consistent with the free radical theory of aging in which the accumulation of ROS and RNS as by-products of aerobic activity in living organisms shortens their lifespan [10,11]. At the cellular level, this oxidative stress can lead to oxidative damage to various macromolecules within the cell, which might lead to cell death and possibly to the death of the organism (Harman, 2003) (Figure 5a). Next, to check our protective antioxidant hypothesis, we evaluate the possible protective effects of AE and CHL at different cellular levels including membrane lipids, proteins and DNA (Figure 5).

First, TBARS assay measuring malondialdehydes (MDA) was used (Figure 5b). MDA, the main degradation products of polyunsaturated lipids induced by ROS, is an oxidative stress biomarker in organisms and can cause damage to membranes [23]. Here, levels of MDA in yeast supplemented by AE and CHL decreased significantly, indicating that both can serve as efficient cell membrane defenses against oxidative stress. Several plant natural products and/or extracts have been reported to reduce MDA production under oxidative stress in yeast [21,23].

Protein carbonyls can form covalent adducts with cellular components that can result in structural alterations and alter their function. Although ROS and RNS are highly reactive and cause site-specific injury, protein carbonyls are more stable and may spread to distant sites, increasing oxidative damage [55]. The protection offered by AE and CHL against UV-C-induced protein carbonylation was demonstrated using the ELISA assay (Figure 5c). The content of carbonyl protein was clearly reduced by returning to levels close to the baseline control level (Figure 5c). It has been shown that the carbonyl groups may result from ROS alteration of the amino acid side chains or reactions with lipid peroxidation products that affect the protein structure [55]. This suggests that AE and CHL could reduce the carbonyl protein content both by scavenging ROS/RNS and by decreasing the lipid peroxidation process. In line with our observation, CHL has been proposed to reduce the protein carbonyl content in human fibroblasts and keratinocytes cells exposed to UV radiation [55].

8-Oxo-Guanine is one of the most common DNA lesions caused by ROS [56]. This oxidative modification of guanine is potentially highly mutagenic, as it may result in uneven pairing with adenine, resulting in substitutions of G to T and C to A in the genome [57]. Protection from AE and CHL supplementation in yeast against UV-C-induced 8-Oxo-Guanine formation was evaluated using the ELISA assay (Figure 5c). As a result of AE and CHL, the 8-Oxo-Guanine content was significantly reduced (Figure 5c). This suggests that AE and CHL could effectively protect DNA from oxidative damage. This has never been seen in vivo in the yeast model to the best of our knowledge. Future work on determining the anti-mutagenic ability of AE and CHL will be undertaken. Overall, our results show that both AE and CHL offer a very complete protection against oxidative stress that can lead to increase in the life span of yeast.

## 4. Conclusions

In this study, using the yeast model, we demonstrate the interest of almond skin extract (AE) and chlorogenic acid (CHL) as new regulators that make a significant contribution to extending the chronological life-span and improving survival under oxidation conditions. This may be linked to their ability to trigger anti-oxidative reactions in yeast during aging and to respond to UV-induced oxidative stress. In both physiological conditions, the AE- and CHL-treated groups showed a reduced production of reactive oxygen and nitrogen species (ROS/RNS). Increased gene expression in *SOD2* as well as of total superoxide dismutase (SOD) activity was recorded during aging in AE and CHL groups. The differential effect at the level of gene expression, with a more pronounced induction of the mitochondrial *SOD2* gene at the expense of the cytosolic *SOD1* gene, indicates the key role of mitochondrial function in this regulation, since normal mitochondrial membrane potential was also maintained during aging in treated groups. Similar activation was also observed for SIR2, an upstream inducer of *SOD2* gene expression and enzymatic activity, thus reinforcing the oxidative protection hypothesis. This role was further confirmed by the use of UV-induced oxidative stress, where AE and CHL improved yeast survival by reducing ROS/RNS production, leading to a significant reduction in cellular oxidative damage as demonstrated by decreased membrane lipid peroxidation, protein carbonyl content and 8-oxo-guanine formation in DNA. This study demonstrates the interest of almonds as a functional food that protects cells from aging and oxidative stress. However, the exact molecular mechanism involved remains unclear, even if the antioxidant activity of CHL can be assumed. In particular, future works with genetic mutants should be conducted to test the causality of the involvement of SIR2 and/or SOD2. The anti-aging effects of AE and CHL should also be confirmed in vivo using different models in future experiments. Their anti-mutagenic potential should also merit future experiments. 

## Supplementary Materials

The following are available online at https://www.mdpi.com/2075-1729/10/6/80/s1, Figure S1: (a) HPLC chromatogram (detection set at 325 nm) of the almond skin extract (*Beldi* genotype grown in the Ain Sfa (34°46′42.4″ N, 002°09′28.9″ W) pilot location in the eastern Morocco) prepared by ultrasound-assisted extraction USAE. **(b)** Structures and corresponding numbers on the HPLC chromatogram of the main phenolic compounds considered in this study: protocatechuic acid (1), *p*-hydroxybenzoic acid (2), chlorogenic acid (3) and *p*-coumaric acid (4). Figure S2: Survival plots used to determine chronological lifespan of yeast (strain DB746) presented in Figure 1a. percentage of viable cells was determined by the microcolony method on YPD plates. Figure S3: Mitochondria integrity estimated by mitochondrial potential (ΔΨm) variation. Table S1: Absolute quantification of chlorogenic acid (and other phenolic acids ^1^) contents in almond skin extract. Table S2: Growth index and viability of yeast cells under the different treatment conditions determined 48h after treatment. Table S3: Estimation of chlorogenic acid and *E*-resveratrol uptake by yeast cell determined 6h after their additions in culture medium. Table S4: Intracellular concentrations of NAD and NADH.
