# Supplementary material for: Almond Skin Extracts and Chlorogenic Acid Delay Chronological Aging and Enhanced Oxidative Stress Response in Yeast
Source: Life (Basel). 2020 May 28;10(6):80. doi: 10.3390/life10060080 (PMC7345664; doi:10.3390/life10060080)
Supplement: Supplementary file 1 [file life-10-00080-s001.pdf]

Supplementary Materials for Almond Skin Extracts and Chlorogenic Acid Delay Chronological Aging and Enhanced Oxidative Stress Response in Yeast

Duangjai Tungmunnithum [1,2,3,§], Malika Abid [4,§], Ahmed Elamrani [4], Samantha Drouet [1,2], Mohamed Addi [4,#,*] and Christophe Hano [1,2,#,*]

[1] Laboratoire de Biologie des Ligneux et des Grandes Cultures, INRAE USC1328, University of Orleans, 45067 Orléans Cedex 2, France; samantha.drouet@univ-orleans.fr (S.D.);

[2] Bioactifs et Cosmetiques, CNRS GDR 3711, 45067 Orléans Cedex 2, France

[3] Department of Pharmaceutical Botany, Faculty of Pharmacy, Mahidol University, Bangkok 10400, Thailand; duangjai.tun@mahidol.ac.th (D.T.);

[4] Laboratoire de Biologie des plantes et des micro-organismes, Faculté des Sciences, Université Mohamed Ier, 60000, Oujda, Maroc; m.abid@ump.ac.ma (M.A.); a.elamrani1@ump.ac.ma (A.E.);

* Correspondence: m.addi@ump.ac.ma (M.Ad.); hano@univ-orleans.fr (C.H.); Tel.: +212 536 500 601 (M.A.); +33-237-309-753 (C.H.);

§ These authors have equal contribution of the first authors.

# These authors have equal contribution of the senior authors.

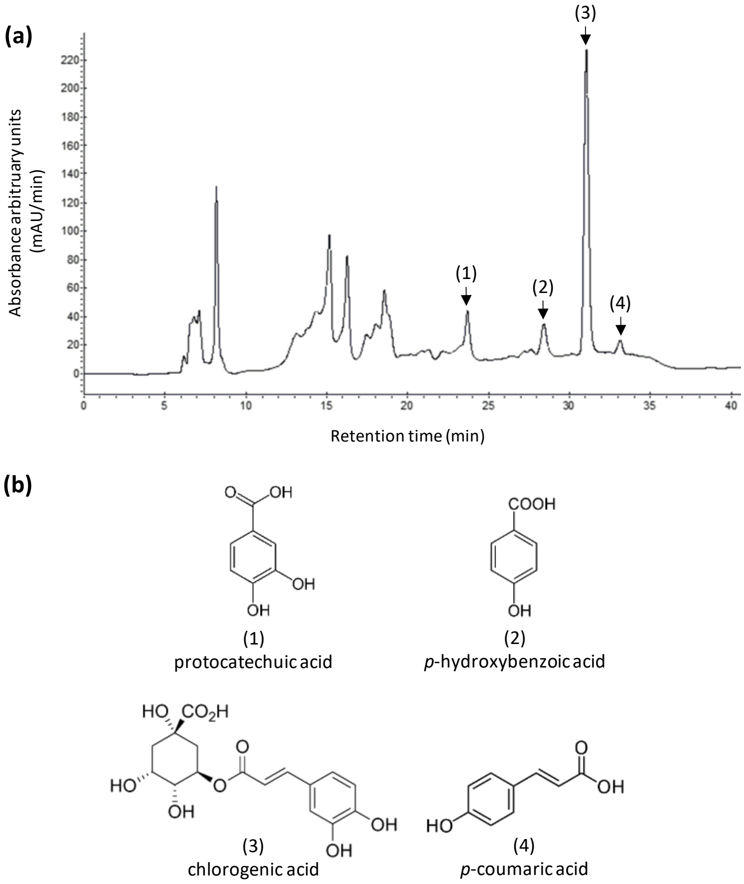

Figure S1. (a) HPLC chromatogram (detection set at 325 nm) of the almond skin extract (*Beldi* genotype grown in the Ain Sfa (34°46′42.4′′N, 002°09′28.9′′W) pilot location in the eastern Morocco) prepared by ultrasound-assisted extraction USAE. **(b)** Structures and corresponding numbers on the HPLC chromatogram of the main phenolic compounds considered in this study: protocatechuic acid (1), *p*-hydroxybenzoic acid (2), chlorogenic acid (3) and *p*-coumaric acid (4).

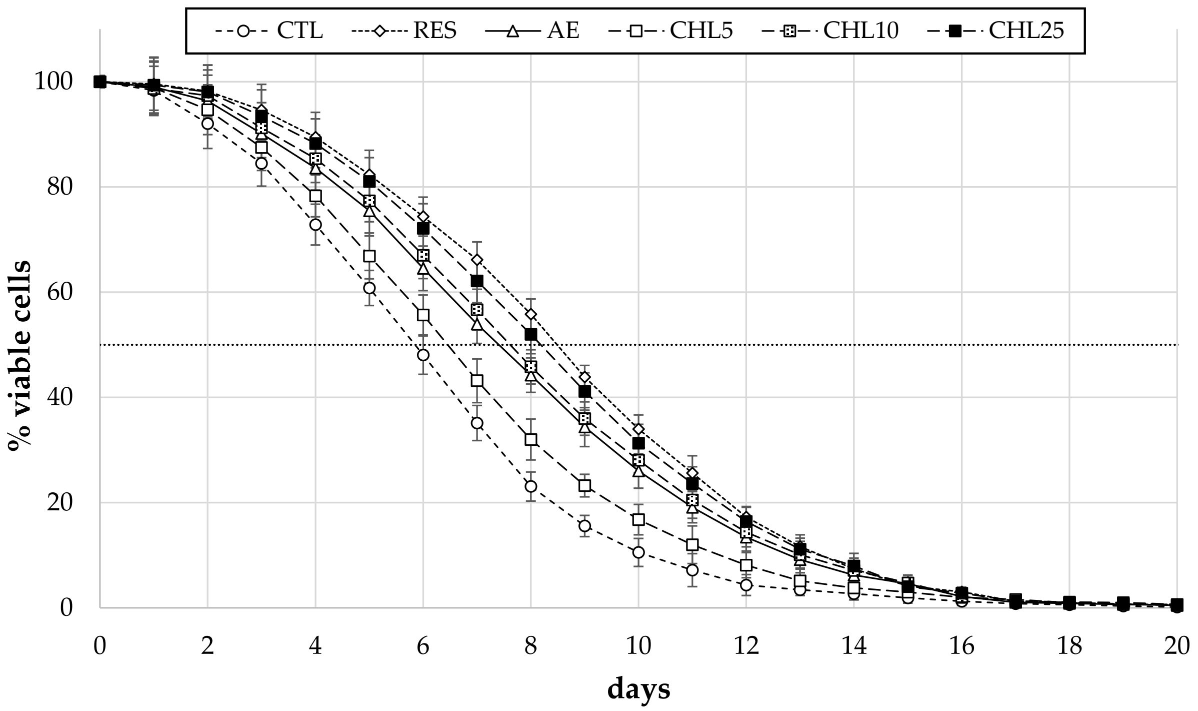

Figure S2. Survival plots used to determine chronological lifespan of yeast (strain DB746) presented in Figure 1a. percentage of viable cells was determined by the microcolony method on YPD plates as described by Hu et al. [27]. Values are means ± standard deviations (SD) of 6 independent experiments.

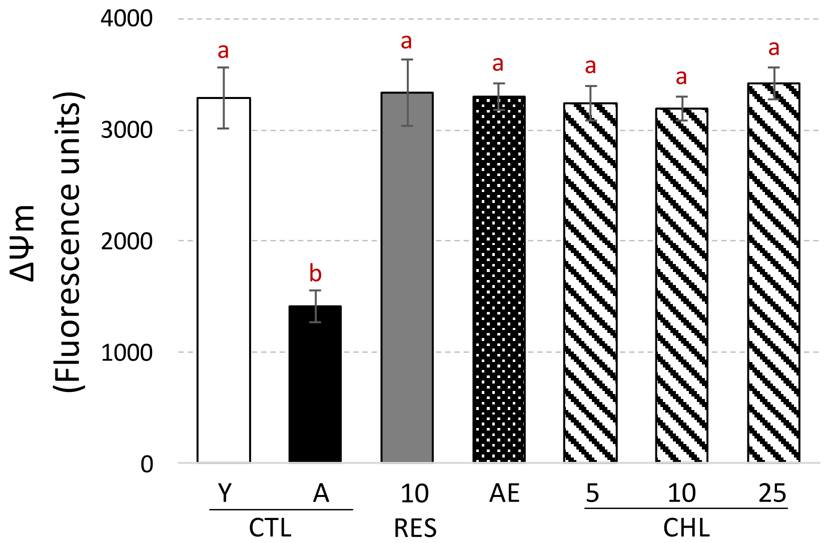

Figure S3. Mitochondria integrity estimated by mitochondrial potential (ΔΨm) variation.

The evaluation of mitochondria membrane potential (ΔΨm) was carried out by treating cells with 3,30-dihexyloxacarbocyanineiodide (DiOC6(3), Sigma-Aldrich, Saint-Quentin Fallavier, France). DiOC6(3) stains mitochondria depending on their ΔΨm. Cells were incubated in culture medium with 25 nM DiOC6(3) for 40 min at 28°C. Results were expressed as relative fluorescent units. Values are means ± SD of 6 independent replicates. Different letters represent significant differences between the different conditions ($p < 0.05$).

Table S1. Absolute quantification of chlorogenic acid (and other phenolic acids) contents in US almond skin extract [2] (in mg/g DW) and final concentration (in μM) applied on yeast.

	TPC[1]	Protocatechuic Acid	*p*-hydroxybenzoic Acid	Chlorogenic Acid	*p*-coumaric Acid
US extract (mg/g DW)	13.86 ± 0.91	2.03 ± 0.07	1.13 ± 0.02	8.14 ± 0.10	0.26 ± 0.20
Concentration applied on yeast (in μM)	88.00 ± 5.78	14.15 ± 0.49	8.83 ± 0.16	25.00 ± 0.30	1.58 ± 1.31

US: ultrasound; [1]TPC: total phenolic content expressed in gallic acid equivalent; other phenolic acids from the extract are: protocatechuic acid, *p*-hydroxybenzoic acid and *p*-coumaric acid contents. [2] *Beldi* genotype grown in the Ain Sfa (34°46'42.4''N, 002°09'28.9''W) pilot location in the eastern Morocco. Values are means ± SD of 3 independent replicates.

Table S2. Growth index and viability of yeast cells under the different treatment conditions determined 48h after treatment.

Conditions	Growth index	Viability
CTL	34.25 ± 1.23 [ab]	95.37 ± 2.61 [a]
RES (10 μM)	34.04 ± 1.72 [ab]	94.27 ± 2.01 [a]
AE	36.12 ± 0.67 [a]	94.45 ± 1.85 [a]
CHL (5 μM)	33.47 ± 0.32 [b]	94.24 ± 1.78 [a]
CHL (10 μM)	33.88 ± 0.19 [b]	96.58 ± 2.92 [a]
CHL (25 μM)	33.38 ± 1.22 [ab]	96.30 ± 3.04 [a]

Almond extract (AE, 1 mg/mL); Chlorogenic acid at 3 concentrations (CHL5, CHL10 and CHL25 corresponding to chlorogenic acid addition at 5, 10 and 25 μM, respectively). *E*-Resveratrol (RES, 10 μM) used as control antiaging drug. Values are means ± standard deviations (SD) of 4 independent experiments. Different letters represent significant differences between the different conditions ($p < 0.05$).

Table S3. Estimation of chlorogenic acid and *E*-resveratrol uptake by yeast cell determined 6h after their additions in culture medium.

Compound	Concentrations/ Conditions	Relative Content in Culture Medium	Relative content in Yeast Cells	Total
E-Resveratrol	10μM	9.43 ± 2.59	80.40 ± 1.81	89.83 ± 4.40
Chlorogenic acid	AE 1	19.20 ± 1.97	70.77 ± 3.56	89.97 ± 5.53
Chlorogenic acid	5 μM	21.10 ± 2.41	70.70 ± 0.53	91.80 ± 2.94
Chlorogenic acid	10 μM	23.47 ± 1.01	71.73 ± 1.47	95.20 ± 2.48
Chlorogenic acid	25 μM	16.60 ± 1.11	74.67 ± 3.90	91.27 ± 5.01

Values are means ± standard deviations (SD) of 4 independent experiments.

Table S4. Intracellular concentrations of NAD and NADH.

Conditions	NAD	NADH	NAD/NADH
CTL	1.56 ± 0.12 [a]	0.93 ± 0.13 [a]	1.70 ± 0.28 [a]
RES (10 μM)	1.59 ± 0.17 [a]	0.98 ± 0.14 [a]	1.63 ± 0.16 [a]
AE	1.46 ± 0.18 [a]	0.84 ± 0.16 [a]	1.77 ± 0.32 [a]
CHL (5 μM)	1.48 ± 0.17 [a]	0.91 ± 0.06 [a]	1.62 ± 0.10 [a]
CHL (10 μM)	1.59 ± 0.15 [a]	0.90 ± 0.19 [a]	1.84 ± 0.48 [a]
CHL (25 μM)	1.62 ± 0.16 [ab]	1.00 ± 0.12 [a]	1.62 ± 0.13 [a]

Values are means ± standard deviations (SD) of 4 independent experiments; expressed in mM for 10^7 cells.

© 2020 by the author. Licensee MDPI, Basel, Switzerland. This article is an open access article distributed under the terms and conditions of the Creative Commons Attribution (CC BY) license (http://creativecommons.org/licenses/by/4.0/).
